# Assessment of the Prevalence and Characteristics of Hip Involvement in Spondyloarthritis: Results From a Rheumatology Outpatient Center

**DOI:** 10.7759/cureus.101822

**Published:** 2026-01-19

**Authors:** Abdellah El Maghraoui

**Affiliations:** 1 Rheumatology, Mohammed V University, Rabat, MAR

**Keywords:** ankylosing spondylitis, coxitis, hip involvement, hips, spondyloarthritis

## Abstract

Aim: Hip involvement is a well-recognized feature of axial spondyloarthritis (AS). This study aimed to assess the prevalence of hip involvement and its relationship with both symptomatic and structural severity in a cohort of AS patients treated in an outpatient rheumatology clinic.

Methods: This retrospective, single-center study was conducted from 2019 to 2023 and included confirmed AS patients. Hip involvement was defined as hip pain related to AS-driven inflammation, confirmed by radiographic imaging (Bath Ankylosing Spondylitis Radiology Hip Index, or BASRI, score of ≥2). Demographic data and parameters of both symptomatic and structural severity were collected. The risk of hip involvement based on disease duration was evaluated using Kaplan-Meier analysis.

Results: Among 137 AS patients, 89 were men (65.0%), and 48 (35.0%) were women. The mean age was 42.2 years (SD ±14, range: 18-76), and the mean disease duration was 7.5 years (SD ±9.1, range: 0.5-38). Radiographic evidence of hip involvement was present in 19 patients (13.9%): unilateral in 9 patients (6.6%) and bilateral in 10 patients (7.3%). Hip involvement was more common in men, those with longer disease duration, and those with a higher incidence of uveitis. Patients with hip involvement exhibited higher symptomatic (Bath Ankylosing Spondylitis Disease Activity Index, or BASDAI, and Ankylosing Spondylitis Disease Activity Score, or ASDAS) and structural (modified Stoke Ankylosing Spondylitis Spine Score, or mSASSS) severity scores. In terms of disease duration, 18.6% of male patients had hip involvement after 10 years, rising to 48.8% after 20 years.

Conclusion: Hip involvement is a frequent manifestation of AS, often presenting bilaterally and associated with longer disease duration and greater disease severity. However, its prevalence appears lower in outpatient settings compared to university hospital series.

## Introduction

Hip involvement is recognized as the primary extraspinal manifestation of axial spondyloarthritis (AS), and it is often linked to poorer functional outcomes. Moreover, hip involvement is a crucial prognostic marker commonly associated with radiographic spinal changes. Studies have reported that between 24% and 36% of AS patients experience hip complications, with 5% of these cases requiring total hip replacement [1-3]. Factors such as increasing age, higher Bath Ankylosing Spondylitis Disease Activity Index (BASDAI) scores, and spinal abnormalities are significant contributors to functional limitations and decreased quality of life [4]. Additionally, there is a strong correlation between the duration of symptoms and hip involvement, suggesting that the longer the disease persists, the more likely patients are to develop severe spinal changes. Various studies have also highlighted the relationship between hip involvement and disease duration [5].

Data from the Prospective Study of Outcomes in Ankylosing Spondylitis (PSOAS) cohort indicates that women with AS may present with a higher incidence of peripheral arthritis, with hip and shoulder involvement, although there is no evidence suggesting more radiographic damage in these areas in women compared to men [6]. On the other hand, numerous studies show that male AS patients exhibit more extensive radiographic damage in the spine compared to female patients, while the differences in hip involvement based on gender remain less studied. Research conducted in North African countries demonstrated that spondyloarthropathies in these regions tend to be more severe than in France, with hip involvement being more frequent [7]. This ethnic variation in the prevalence of hip involvement was further corroborated by studies conducted in France [8].

The purpose of this study was to examine the prevalence of hip involvement among AS patients receiving care in an outpatient rheumatology clinic, to assess the radiological prevalence of hip involvement among Moroccan AS patients, to evaluate patient characteristics and clinical assessments related to this condition, and to identify any potential gender differences.

## Materials and methods

Study population

The patients included in this retrospective study were all diagnosed with AS and were followed up at a private rheumatology outpatient clinic in Rabat, Morocco, over a four-year period from 2019 to 2023. The inclusion criteria required patients to meet the modified Assessment of Spondyloarthritis International Society (ASAS) criteria for AS and to be at least 18 years old. This study adhered to the principles outlined in the Declaration of Helsinki. In Morocco, retrospective studies involving patient charts do not require notification or approval from Regional Ethics Committees.

Data collection

Patient data was collected retrospectively from patients' medical records, which included medical history, laboratory findings, and radiographic imaging of the spine and pelvis. The information gathered consisted of demographic details (sex, age), family history of AS (in first-degree relatives), symptom duration, disease duration (time since diagnosis), and the presence of peripheral arthritis (excluding the hip), inflammatory bowel disease (IBD), psoriasis, and uveitis. Disease activity was assessed using the Bath Ankylosing Spondylitis Disease Activity Index (BASDAI) and Ankylosing Spondylitis Disease Activity Score (ASDAS), which were calculated based on C-reactive protein levels.

Radiographic assessment

Radiographs were evaluated by the author, who had extensive expertise in the relevant scoring indexes. Sacroiliitis and hip involvement were assessed using anteroposterior pelvic X-rays and graded according to the New York criteria and Bath Ankylosing Spondylitis Radiology Hip Index (BASRI). Hip involvement was defined as a radiographic BASRI hip score of ≥2. The mean BASRI hip score was calculated when the hip involvement was bilateral. Spinal involvement was scored using the modified Stoke Ankylosing Spondylitis Spine Score (mSASSS), and lumbar spine syndesmophytes were evaluated using standard posteroanterior and lateral lumbar spine radiographs.

Statistical analysis

The prevalence of hip involvement was measured, and comparisons between patients with and without hip involvement were conducted using the Mann-Whitney U test for quantitative variables and the chi-square test for qualitative variables. We compared male and female patients, utilizing the Student’s t-test for quantitative variables and the chi-square test for qualitative variables. Lastly, the risk of hip involvement was estimated in relation to disease duration using the Kaplan-Meier method. The onset of hip involvement was defined as the date when the first clinical symptoms of hip disease appeared, while the onset of AS was marked by the first symptoms of spondyloarthritis.

## Results

A total of 137 patients with AS were evaluated, consisting of 89 men (65.0%) and 48 (35.0%) women. Their main characteristics are summarized in Table 1. The mean age of the participants was 42.2 years (SD ±14, range: 18-76), and the mean disease duration was 7.5 years (SD ±9.1, range: 0.5-38). Extra-articular manifestations were present in 55 patients (40.1%), including uveitis in 25 (18.2%), inflammatory bowel disease in 15 (10.9%), and psoriasis in 15 (10.9%). Sacroiliitis was confirmed by X-ray in 87 patients (63.5%) and by MRI in 28 patients (20.4%). Of the 70 patients tested, 52 (74.3%) were HLA-B27 positive. Throughout the course of the disease, 48 patients (35.0%) received treatment with tumor necrosis factor-alpha (TNF-α) inhibitors at some point.

**Table 1:** Characteristics of the study population mSASSS: modified Stoke Ankylosing Spondylitis Spine Score; BASRI: Bath Ankylosing Spondylitis Radiology Hip Index; BASDAI: Bath Ankylosing Spondylitis Disease Activity Index; ASDAS: Ankylosing Spondylitis Disease Activity Score; ESR: erythrocyte sedimentation rate; CRP: C-reactive protein; IBD: inflammatory bowel disease; MRI: magnetic resonance imaging ^*^Indicates a statistically significant result.

	Total, N=137	Male patients, n=89	Female patients, n=48	p
Age (years), M (SD)	42.2 (14.0)	41.3 (14.3)	43.8 (13.5)	0.319
Disease duration (years), M (SD)	7.5 (9.1)	7.6 (9.4)	7.2 (8.7)	0.777
mSASSS, M (SD)	3.6 (11.4)	3.3 (9.0)	4.4 (15.7)	0.655
ESR (mm/hr), M (SD)	20.6 (21.3)	17.9 (21.2)	25.7 (20.3)	0.076
CRP (mg/l), M (SD)	10.5 (21.5)	12.4 (25.4)	6.9 (6.1)	0.090
HLA-B27, n (%)	52/70 (74.3 %)	39/50 (78.0%)	13/20 (65.0%)	0.411
BASDAI, M (SD)	27.3 (23.0)	29.7 (25.3)	22.0 (15.9)	0.093
ASDAS, M (SD)	2.0 (1.7)	2.1 (1.9)	1.6 (0.7)	0.077
Uveitis, n (%)	25 (18.2)	18 (20.5)	7 (14.6)	0.539
Psoriasis, n (%)	15 (10.9)	8 (9.1)	7 (14.6)	0.477
IBD, n (%)	15 (10.9)	13 (14.6)	3 (6.3)	0.626
Sacroilitis on X-rays, n (%)	87 (63.5)	53 (59.6)	34 (70.8)	0.335
Sacroilitis on MRI, n (%)	28/30 (93.3)	17/17 (100.0)	11/13 (84.3)	0.875
Hip involvement, n (%)	19 (13.9)	16 (17.9)	3 (6.3)	0.046*
BASRI hip, M (SD)	0.4 (1.2)	0.5 (1.4)	0.1 (0.7)	0.037*
Biologics use, n (%)	48 (35.0)	32 (35.9)	16 (33.3)	0.905

Radiographic evidence of hip involvement was detected in 19 patients (13.9%). Of these, 9 patients (6.6%) had unilateral hip involvement, while 10 patients (7.3%) had bilateral hip involvement. The average BASRI hip score was 0.4 (±1.2). A comparison of patients with and without hip involvement revealed that those with hip involvement were predominantly male, had longer disease durations, and exhibited a higher prevalence of uveitis (Table 2). Additionally, these patients had higher scores for symptomatic (BASDAI and ASDAS) and structural (mSASSS) AS severity.

**Table 2:** Comparison between patients with and without hip involvement mSASSS: modified Stoke Ankylosing Spondylitis Spine Score; BASRI: Bath Ankylosing Spondylitis Radiology Hip Index; BASDAI: Bath Ankylosing Spondylitis Disease Activity Index; ASDAS: Ankylosing Spondylitis Disease Activity Score; ESR: erythrocyte sedimentation rate; CRP: C-reactive protein; IBD: inflammatory bowel disease; MRI: magnetic resonance imaging *Indicates a statistically significant result.

	Patients with hip involvement, n=19	Patients without hip involvement, n=118	p
Age (years), M (SD)	46.9 (17.7)	41.4 (13.3)	0.210
Gender, male, n (%)	16 (84.2)	73 (61.8)	0.046*
Disease duration (years), M (SD)	11.3 (8.9)	6.9 (9.1)	0.003*
HLA-B27, n (%)	9/10 (90.0)	43/60 (71.7)	0.402
Uveitis, n (%)	25 (21.2)	0 (0)	0.042*
Psoriasis, n (%)	1 (5.2)	14 (11.8)	0.153
IBD, n (%)	3 (15.9)	13 (11.0)	0.494
Sacroilitis on X-rays, n (%)	16 (84.2)	71 (61.2)	0.042*
Sacroilitis on MRI, n (%)	1/1	27/29	1.0
mSASSS, M (SD)	12.6 (22.3)	2.1 (7.7)	<0.001*
ESR (mm/hr), M (SD)	20.7 (16.2)	20.7 (22.3)	0.973
CRP (mg/l), M (SD)	17.7 (40.9)	9.1 (14.6)	0.404
BASDAI, M (SD)	49.2 (25.3)	25.3 (20.2)	<0.001*
ASDAS, M (SD)	2.6 (1.0)	1.9 (1.7)	0.002*
Biologics use, n (%)	10 (52.6)	38 (32.2)	0.140

**Figure 1:**
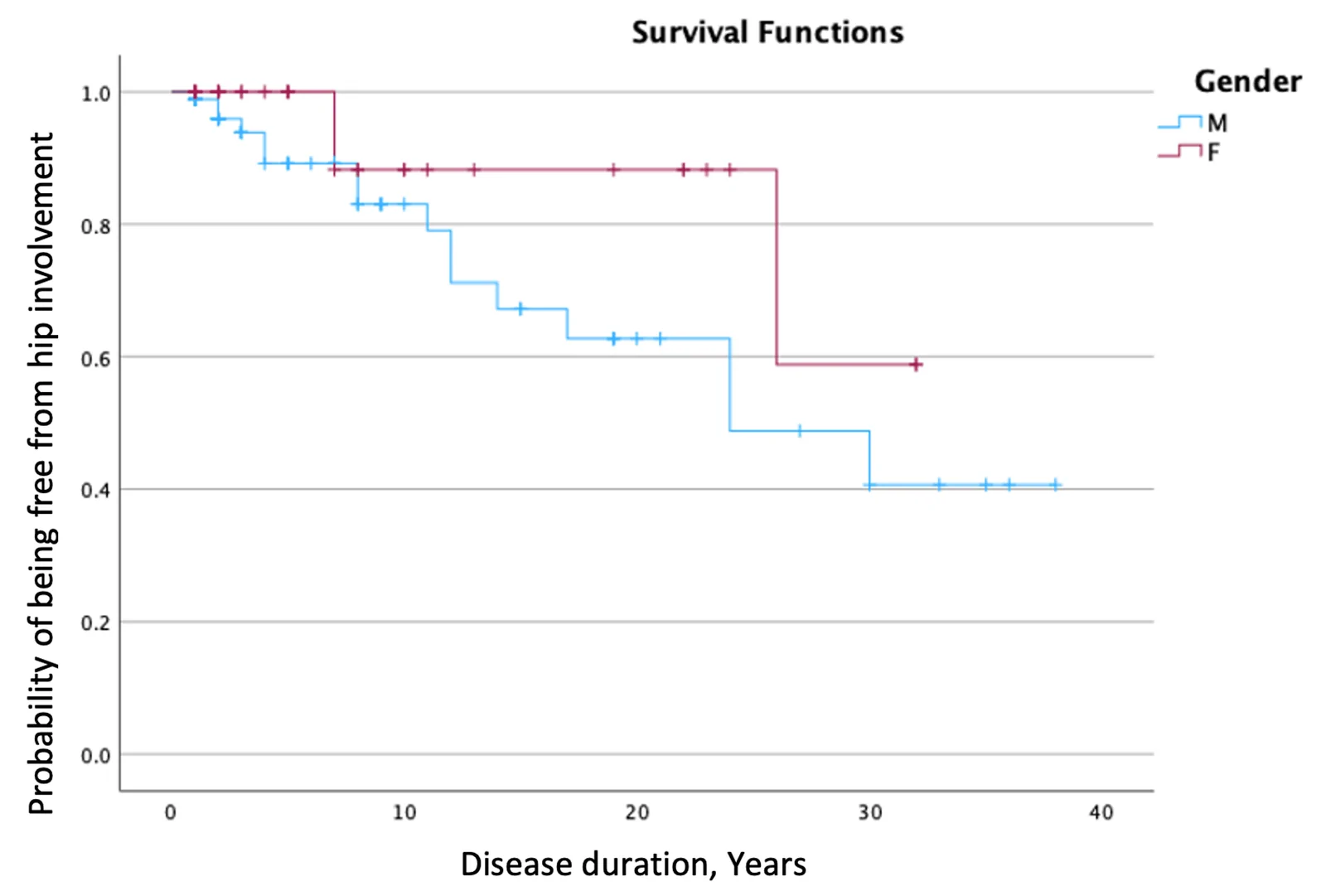
Survival curve of hip involvement probability in male and female patients with axial spondyloarthritis M: males; F: females

The risk of hip involvement, as estimated in relation to disease duration using the Kaplan-Meier analysis (Figure 1), showed that hip involvement developed in 18.6% of male patients after 10 years, in 48.8% after 20 years, and in 58.8% after 30 years.

## Discussion

Compared to spinal involvement, hip involvement in AS has received less attention in research, with most studies coming from university hospital settings. In this retrospective study of AS patients from an outpatient rheumatology clinic, hip involvement was found in 13.9% of cases, with the prevalence increasing with disease duration (up to 30% after 20 years). Patients with hip involvement exhibited more severe symptomatic and structural disease. The involvement was frequently bilateral, and, in some cases, appeared early in the disease course, with a median onset of two years. TNF-α inhibitors were prescribed at least once in 52% of patients with hip involvement.

The prevalence observed in this study aligns with findings from other outpatient clinic-based studies. For example, a study conducted at the Samsung Medical Center, Seoul, South Korea, reported hip arthritis in 12.3% of AS patients (60 out of 488) [9]. Similarly, a Taiwanese study found radiographic hip involvement in 9% (48 out of 531) of TNF inhibitors (TNFi)-naïve AS patients [10]. However, higher rates of hip involvement have been reported in university hospital cohorts and tertiary care facilities, with prevalence ranging from 24% to 36%. A multicenter study conducted across Moroccan university rheumatology departments indicated the hip involvement prevalence rates to range from 19% to 41% [11], while Tunisian studies reported rates between 29% and 36% [12,13]. Research groups from Belgium (ASPECT), Spain (REGISPONSER), and Ibero-America (RESPONDIA) found similar rates, ranging from 24% to 36% [1,14].

In a single-center French study, hip involvement was identified in 18% of AS patients (49 out of 275) [8]. Notably, 40% of North African patients in this study exhibited hip involvement, compared to just 16% of Caucasians (p=0.014), suggesting that ethnicity may influence the risk of hip involvement. Previous studies using multivariate analyses have suggested that environmental factors may also play a significant role in the disease’s progression [15].

There is no universal standard for defining hip involvement in AS. Diagnosis typically relies on patient-reported symptoms, physical examinations (e.g., hip rotation, intermalleolar distance), and radiographic imaging. This lack of standardization can result in varying prevalence rates. In this study, there was a slight predominance of male patients with hip involvement, which is consistent with the study findings of Burki et al. [8]. However, other studies, including the PSOAS cohort in Taiwan [6], those from China [5] and Korea [9], as well as others, have reported no significant gender differences in hip involvement. Additionally, sacroiliitis was more frequent among patients with hip involvement, likely reflecting more severe disease. These patients were also more likely to use TNF-α inhibitors.

In our study, 52% of patients with hip involvement were treated with TNF-α inhibitors, which aligns with previous studies. One recent study suggested that TNF inhibitors may help stabilize radiographic damage in the hips, even as spinal damage continues to progress [14]. It is estimated that approximately 49% of AS patients (ranging from 37% to 78%) are candidates for TNF-α therapy due to high disease activity, functional impairment, and hip involvement.

This study was not without limitations. One potential bias stems from the fact that all patients were drawn from a single outpatient clinic, which may have led to the recruitment of less severe cases. Additionally, the onset of hip pain was based on patient recall, which could introduce inaccuracies. Despite its limitations, this study has several strengths, including the assessment of patients from a real-world private rheumatology setting without selection based on disease severity, the use of standardized clinical and radiographic evaluations, and the availability of detailed clinical data that allowed for characterization of hip involvement.

## Conclusions

In conclusion, hip involvement in axial spondyloarthritis is a significant and severe complication, frequently associated with prolonged disease duration and greater structural and symptomatic severity. The prevalence of hip involvement in this outpatient cohort was 13.9%, which is notably lower than rates reported by university hospital studies, likely reflecting differences in patient populations. Factors such as male gender, longer disease duration, and associated uveitis were identified as key predictors of hip involvement, emphasizing its relevance as a prognostic marker. This study underscores the importance of early detection and management of hip involvement, particularly through the use of targeted biological therapies to improve long-term outcomes. Future research should aim to better understand the interplay between demographic, clinical, and environmental factors in hip involvement to refine treatment strategies and reduce its impact on quality of life.
